# Use of Polyphenol Tannic Acid to Functionalize Titanium with Strontium for Enhancement of Osteoblast Differentiation and Reduction of Osteoclast Activity

**DOI:** 10.3390/polym11081256

**Published:** 2019-07-29

**Authors:** Chris Steffi, Zhilong Shi, Chee Hoe Kong, Sue Wee Chong, Dong Wang, Wilson Wang

**Affiliations:** Department of Orthopaedic Surgery, National University of Singapore, NUHS Tower Block Level 11, 1E Kent Ridge Road, Singapore 119228, Singapore

**Keywords:** osteoporosis, strontium, polyphenol tannic acid, titanium, osteoblasts, osteoclasts

## Abstract

Implant anchorage remains a challenge, especially in porous osteoporotic bone with high osteoclast activity. The implant surface is modified with osteogenic molecules to stimulate osseointegration. Strontium (Sr) is known for its osteogenic and anti-osteoclastogenic effects. In this study, Sr was immobilized on a titanium (Ti) surface using bioinspired polyphenol tannic acid (pTAN) coating as an ad-layer (Ti-pTAN). Two separate coating techniques were employed for comparative analysis. In the first technique, Ti was coated with a tannic acid solution containing Sr (Ti-pTAN-1Stp). In the second method, Ti was first coated with pTAN, before being immersed in a SrCl_2_ solution to immobilize Sr on Ti-pTAN (Ti-pTAN-2Stp). Ti-pTAN-1Stp and Ti-pTAN-2Stp augmented the alkaline phosphatase activity, collagen secretion, osteocalcin production and calcium deposition of MC3T3-E1 cells as compared to those of Ti and Ti-pTAN. However, osteoclast differentiation of RAW 264.7, as studied by TRAP activity, total DNA, and multinucleated cell formation, were decreased on Ti-pTAN, Ti-pTAN-1Stp and Ti-pTAN-2Stp as compared to Ti. Of all the substrates, osteoclast activity on Ti-pTAN-2Stp was the lowest. Hence, an economical and simple coating technique using pTAN as an adlayer preserved the dual biological effects of Sr. These results indicate a promising new approach to tailoring the cellular responses of implant surfaces.

## 1. Introduction

Low bone mineral density and altered microarchitecture are prominent characteristics of osteoporotic bone, especially in post-menopausal, elderly women [1]. Apart from being susceptible to fractures, the porous architecture of fragile osteoporotic bones affects the anchorage of screws, leading to post-surgical complications and implant failures [2]. Balanced regulation of osteoblast and osteoclast activity in the peri-implant region is crucial for successful osseointegration [3,4]. Thus, the surfaces of implants were functionalized with osteogenic molecules to promote bone-implant integration [5,6]. The physiochemical properties of the implant surface, such as roughness, topography and chemical modifications, play a crucial role in modulating the activity of bone cells at the bone-implant interface, and hence, influence osseointegration [7,8,9]. Surfaces were also functionalized with bone morphogenetic protein-2 (BMP-2), BMP-7, vascular endothelial growth factor and hydroxyapatite to regulate the cellular behavior on the implant surface and augment ossteointegration [6,10,11,12]. Anti-osteoporotic drugs, such as bisphosphonates, have gained research interest in surface science by demonstrating an improvement of bone-implant integration in osteoporotic rats [13]. Yet, atypical fractures caused by bisphosphonates remain a concern [14]. 

Strontium ranelate was shown to augment bone mineral density and reduce the risk of fragility fractures in post-menopausal, elderly women [15,16]. Along with long-term beneficial effects, the medication was cost-effective and was approved in Europe for osteoporosis treatment [16]. Oral administration of strontium ranelate to ovariectomized rats reduced osteoporosis-induced bone loss, while bone quality was preserved [17]. At a cellular level, the effects of Sr are cell specific. For instance, the differentiation of osteoblasts improved with strontium ranelate, whereas osteoclast activity was impeded by strontium ranelate treatment [18]. These established the suitability of the dual actions of strontium (Sr) for osteoporotic bone. Apart from oral administration, research has been carried out to immobilize Sr on implant surfaces [19]. A titanium surface was functionalized with strontium-substituted hydroxyapatite (HA) by electrochemical deposition, which boosted implant integration in osteopenic rats [20]. In another study, a Ti surface modified with Sr-loaded nanotubules promoted new bone formation and improved bone-implant contact in an in vivo rat model [21]. In a comparative analysis, HA, substituted with three different osteogenic metals, i.e., zinc, magnesium and strontium, was coated onto a Ti surface by electrochemical deposition [22]. All metal-HA substitutions revealed improved osseointegration in osteopenic rats as compared to that of unsubstituted-HA, with the osteopromotive effect of Sr being the highest of all. Because of the osteopromotive effects of Sr, in this study, Sr was shortlisted to functionalize Ti via an economical and facile coating technique using a bioinspired polyphenol coating. 

Surface modification of bone implants is evolving from coating with passive materials having a composition similar to bone to the immobilization of active compounds via coatings which trigger desired biological responses and result in rapid and permanent implant fixations within native bone. Recently, bioinspired coatings were achieved using natural polyphenols such as polyphenol tannic acid (pTAN) [23]. These molecules oligomerized by successive autooxidation reactions. Thus, the formed oligomers have reduced solubility and a high affinity for surfaces on which to be deposited. Polyphenols form complexes with metal ions, providing the stability that is dependent on pH and valency states of the metal ions [24]. For example, metals ions (sodium) from a reaction buffer used for the coating were detected in the surface coatings of pTAN. The concentration of these metal ions influences the kinetics of pTAN coating, suggesting that apart from oxidation reactions, metal ion coordination bonds are involved in the coating process [25]. In a separate study, pTAN was shown to coat different surfaces rapidly by forming pH-dependent coordination bonds with Fe(III) ions [26]. Apart from the ability of pTAN to interact with metal ions, pTAN coatings possess anti-bacterial and anti-oxidant properties [23]. Moreover, U.S. Food and Drug Administration classifies tannic acid as generally regarded as safe (GRAS). These findings outline the advantages of using pTAN as an adlayer. 

Consequently, this study exploited the metal binding capability of pTAN coating to immobilize Sr^2+^ on Ti. The potency of the immobilized Sr was monitored by studying osteoblast and osteoclast differentiation. Sr immobilized substrates were studied for osteoblasts markers such as alkaline phosphatase (ALP) activity, collagen type 1 (COLL 1), osteocalcin and calcium deposition. Osteoclast markers such as tartarate resistance acid phosphatase (TRAP) activity, total DNA, and multinucleated cell formation were also assessed on the surface coatings. 

## 2. Materials and Methods

### 2.1. Materials

All chemicals obtained from Sigma-Aldrich (Darmstadt, Germany) were utilized as received. Chemicals and reagents purchased elsewhere are mentioned in the text. All experiments used milli-Q water (>18.2 MΩ·cm) unless otherwise stated (ultrapure water system, Arium 611UF, Sartorius Stedim Biotech GmbH, Göttingen, Germany).

### 2.2. Material Preparation

Sandpaper (600- and 1200-grit) was used to polish titanium alloy (Ti-6Al-4V foils, 10 mm × 10 mm, Goodfellow Cambridge Ltd. Huntingdon, England). The titanium (Ti) foils were then rinsed in water-bath ultrasonicator for 10 min. Sonication was further carried out in Kroll′s reagent (4.0% HF, 7.2% HNO_3_, 88.8% water) to remove the accumulated carbide resulted from polishing [10]. Next, the reaction was stopped by 1 N sodium hydroxide addition, and subsequently, the substrates were sonicated 10 min each in dichloromethane, acetone, and water. The substrates were immersed for 30 min in 40% nitric acid for surface passivation and thoroughly washed with water to remove nitric acid. 

The procedure for polyphenol tannic acid (pTAN) coating for Ti (named as Ti-pTAN) was followed as previously reported with slight modification [23]. In brief, Ti foils were immersed for 24 h in 2 mg/mL solution of pTAN prepared in bicine buffer (100 mM) with 0.6 M NaCl at 7.8 pH. The procedure was carried out in dark with gentle shaking. The substrates were washed thoroughly in water and further submerged in water for 2 days at 37 °C; the water was changed every 24 h.

**Figure 1:**
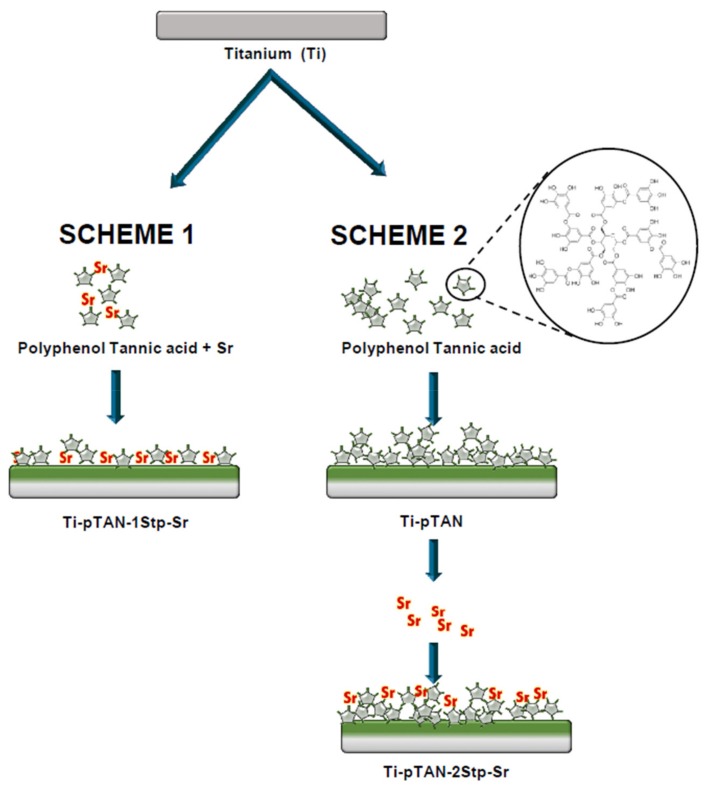
Schematic representation of the workflow of Ti-pTAN-1Stp-Sr and Ti-pTAN-2tp-Sr coatings.

Ti-pTAN substrates were functionalized with Sr by two different methods (Figure 1). In the first one-step method, Ti foils were incubated in pTAN solution (2 mg/mL, 100 mM bicine buffer, pH = 7.8) containing SrCl_2_ (0.3 M Sr) for 24 h with gentle shaking in dark. 0.3 M SrCl_2_ was used to maintain the similar ionic stoichiometry as 0.6 M NaCl in bicine buffer. These substrates were named Ti-pTAN-1Stp. The second method was a 48 h-long, two-step process wherein pTAN coating and Sr coating were carried out separately. In the first 24 h, Ti was coated with pTAN, as discussed above. Subsequently, Ti-pTAN substrates were immersed in a SrCl_2_ solution (0.1 M Sr in milli-Q water) for another 24 h in the dark. In this case, the Sr solution comprising minimum Sr concentration with maximum immobilisation efficiency was used. These substrates were denoted as Ti-pTAN-2Stp. After the Sr coating, Ti-pTAN-1Stp and Ti-pTAN-2Stp were sonicated for 5 min and washed thoroughly with milli-Q water to remove unbound Sr. The substrates were irradiated with UV for 30 min and transferred to a 24-well cell culture plate prior to cell seeding.

### 2.3. Substrate Characterization

Surface chemical composition of all the substrates were analyzed using X-ray photoelectron spectroscopy (XPS) (Kratos AXIS Ultra DLD spectrometer, Kratos Analytical Ltd, Manchester, UK). The binding energies of all the elements were referenced to C 1s signal at 284.6 eV. The surface roughness was studied using a contact based Nanomap-LS 3D profilometer (AEP Technologies, Santa Clara, CA, USA). Water contact angle (WCA) analysis for all the substrates were performed using contact angle analyzer Phoenix 300 touch (Surface Electro Optics, Gyeonggi-do, Korea). The measurements were carried out according to the sessile drop technique and a video camera. Ten microliters of sessile drop of distilled water was transferred onto the substrate using a micro-syringe. The contact angle values on both sides of the drop were measured using tangent rule to ensure symmetry and horizontal level. To estimate the amount of bound Sr ions, Ti-pTAN-1Stp and Ti-pTAN-2Stp substrates were soaked in a 2% nitric acid solution overnight with shaking. Sr ions dissolved in the nitric acid solution were quantified using inductively coupled plasma mass spectrometry (ICP-MS, CX 7700x Agilent, Santa Clara, CA, USA).

### 2.4. Strontium Ions Release Study

Ti-pTAN-1Stp and Ti-pTAN-2Stp substrates were immersed in 1 mL of phosphate buffer saline (PBS) at 37 °C for 14 days. PBS was collected at regular intervals and replenished with fresh PBS. Sr ions in released in PBS were measured using inductively coupled plasma mass spectrometry (ICP-MS, CX 7700x Agilent, CA, USA). 

### 2.5. Cell Culture

Mouse pre-osteoblasts MC3T3-E1 (ATCC, Manassa, VA, USA) were grown in complete Minimum Essential Medium Alpha (MEM-α, 10% fetal bovine serum (FBS), 100 U/mL penicillin and 100 μg/mL streptomycin (Thermo Fisher Scientific, Waltham, MA, USA). Osteoblast differentiation media was prepared by adding 10 mM sodium β-glycerophosphate and 50 μg/mL ascorbic acid to complete phenol red free MEM-α. Then, 40,000 cells were seeded per substrate placed in a 24-well plate. 

RAW 264.7 (ATCC, Manassa, VA, USA) murine pre-osteoclasts were cultured in complete Dulbecco′s Modified Eagle Medium (10% heat inactivated FBS, 100 U/mL penicillin and 100 μg/mL streptomycin (Invitrogen, Waltham, MA, USA). For osteoclast differentiation studies, RAW 264.7 were cultured in presence of 50 ng/mL murine RANKL (R&D Systems, Minneapolis, MN, USA) in complete phenol red free MEM-α (10% heat inactivated FBS, 100 U/mL penicillin, 100 μg/mL streptomycin) at cell density of 2000/cm^2^ on all the substrates. 100 μL of cell suspension was loaded on the substrates. After 6 h of incubation, an additional 400 μL of cell culture media was added to the wells. Cells were maintained in a humid environment at 37 °C with 5% CO_2_, and the spent media were changed every 2–3 days.

### 2.6. MC3T3-E1 Proliferation

Cell proliferation of MC3T3-E1 on all the substrates was assessed on day 1, day 4, day 7 and day 14. Cells were incubated in Cell Counting Kit-8 (CCK-8, Dojindo Laboratories, Kumamoto, Japan) working reagent, prepared by mixing CCK-8 in complete MEM-α (1:10 *v*/*v*). After 4 h, the absorbance was measured at 450 nm by microplate reader (Synergy H1, BioTek Instruments, Inc. Winooski, VT, USA). 

### 2.7. ALP Activity, Collagen-1 and Osteocalcin Production of MC3T3-E1 Cells

ALP activity of osteoblasts was estimated in the cell culture supernatant at various time points with QuantiChrom Alkaline Phosphatase Assay Kit (BioAssay Systems, Hayward, CA, USA). In brief, 50 μL of sample was incubated with 150 μL ALP working reagent for 4 min. Absorbance of 405 nm was measured at 0 and 4 min using microplate reader (Synergy H1). The ALP activity in terms of pNP produced was estimated as per the formula provided in the kit, and normalized with the total protein. The unit for ALP was μM of pNP produced per minute per mg of protein. Collagen 1 and osteocalcin were also measured in cell culture supernatant on day 14 by using Pro-Collagen I alpha 1 SimpleStep ELISA kit (Abcam, Cambridge, UK) and Osteocalcin ELISA kit (Cloud-Clone Corp., Katy, TX, USA) respectively, and the values were normalized to total protein.

### 2.8. Collagen Staining of Osteoblasts

MC3T3-E1 was cultured on various substrates for 14 days. After washing with phosphate buffer saline (PBS), cells were fixed for 15 min in 4% paraformaldehyde which was followed by 5 min treatment with 0.1% triton X-100 to permeabilise cell membrane. Non-specific binding was blocked by overnight incubation of cells in 3% bovine serum albumin (BSA) at 4 °C. Cells were incubated with anti-mouse collage type I primary antibody (Merck, Darmstadt, Germany) for 90 min. Secondary antibody Alexa Fluor 546 tagged anti-rabbit IgG (H + L) (Invitrogen) and alexa Fluor 488 phalloidin (Invitrogen) for actin staining were added to cells for 30 min followed by nuclei labelling using DAPI. Cells images were acquired using Olympus FV1000 confocal laser scanning microscope (CSLM, Olympus, Tokyo, Japan).

### 2.9. Alizarin Staining and Calcium Estimation

After 14 days of culture MC3T3-E1 cells in osteogenic medium, the cells were washed with PBS and fixed with 70% ethanol at 4 °C for 1 h. Alizarin red solution (2% in water) was added to the cells for 1 h. The cells were washed with copious amount of water to remove unbound alizarin and observed under microscope (Leica Microsystems, Wetzlar, Germany). 

Calcium deposited by MC3T3-E1 cells was also quantified after 14 days of culture. The substrates were submerged in 500 μL of 2% nitric acid overnight with gentle shaking, to dissolve the deposited calcium. The eluted calcium was then quantified using QuantiChrom Calcium Assay Kit (BioAssay Systems, Hayward, CA, USA) according to manufacturer′s instructions. In brief, 5 μL of the sample was incubated with 200 μL of kit′s working solution for 5 min. Absorbance was measured at 612 nm using a microplate reader (Synergy H1). 

### 2.10. TRAP Activity

To trigger osteoclastogenesis, RAW 264.7 cells were exposed to 50 ng/mL of RANKL for 3 and 5 days. TRAP activity of the cells was estimated in cell culture supernatant at day 3 and day 5 of culture using TRAP activity assay kit (Takara Bio Inc, Shiga, Japan). Sample was mixed with acid phosphatase buffer (1:1 *v*/*v*) provided with the kit and incubated for 60 min at 37 °C. Stop solution, 0.5 N sodium hydroxide, was added and absorbance was taken at 405 nm by microplate reader (Synergy H1). The calculation of TRAP activity was based on pNP generated, which were normalized to total protein. Hence, the unit of TRAP activity was µM of pNP produced per minute per mg of protein.

### 2.11. DNA Quantification

RAW 264.7 cells were lysed by three freeze thaw cycles in milli-Q water following 5 days of culture with RANKL. After centrifugation for 20 min at 12,000 g (4 °C), the samples were mixed with picogreen reagent (Thermo Fisher Scientific, Waltham, MA, USA) and incubated for 5 min at RT. A microplate reader (Synergy H1) was used to excite the samples at 480 nm, and emission was measured at 520 nm. 

### 2.12. Actin and TRAP Staining of Osteoclasts

RAW 264.7 cells were cultured on various substrates. After 5 days of RANKL exposure, the cells were fixed with 4% paraformaldehyde for 15 min and incubated for 5 min in triton 0.1% X-100 to increase cell membrane permeability. To prevent non-specific binding, the cells were immersed in 3% BSA (overnight, 4 °C). 90 min incubation in anti-TRAP primary antibody (Takara Bio Inc, Shiga, Japan) followed by 30 min incubation in premixed solution of secondary antibody (Alexa Fluor 546 tagged-Goat anti-Mouse IgG (H + L), Thermo Fisher Scientific) and phalloidin (Alexa fluor 488 tagged, Thermo Fisher Scientific) was carried out to stain TRAP and actin in the cells. After nuclei staining with DAPI, the cells were imaged with Olympus FV1000 CLSM (Olympus, Japan). Multinucleated cells with more than three nuclei (blue), and positive for TRAP (red) were classified as osteoclasts. 

### 2.13. Statistics

All experiments were replicated more than three times and the data were presented as mean ± standard deviation (SD). The statistical analysis was carried out by one-way analysis-of-variance and Tukey post hoc test. *p* < 0.05 was considered as statistically significant.

## 3. Results

### 3.1. Surface Characterization

**Figure 2:**
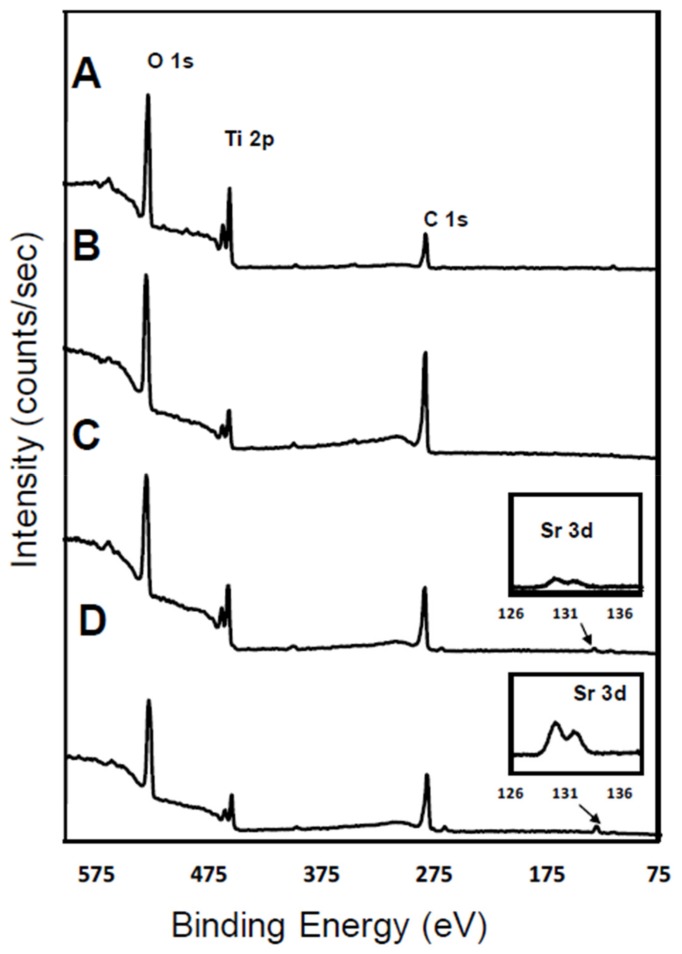
XPS wide spectra scan of (A) Ti; (B) Ti-pTAN; (C) Ti-pTAN-1Stp-Sr; (D) Ti-pTAN-2Stp-Sr.

In the current study, polyphenol tannic acid chemistry is utilized to immobilize Sr on a Ti surface. The elemental compositions of the surfaces were examined by XPS. Signal peaks for elements such as O 1s (530 eV), Ti 2p (460 eV) and C 1s (285 eV) were observed on pristine Ti (Figure 2A and Table 1). C 1s signal on Ti was present due to inevitable hydrocarbon contamination. Therefore, C 1s signals are commonly used as reference for signal calibrations in XPS scans [27]. Ti-pTAN substrates displayed a decrease in Ti 2P signal signifying successful deposition of pTAN on Ti (Figure 2B and Table 1). Ti-pTAN-1Stp and Ti-pTAN-2Stp also displayed a drop in Ti 2P signals. However, Ti 2P signals on Ti-pTAN-1Stp was higher than the Ti 2P signals on Ti-pTAN and Ti-pTAN-2Stp. This suggested that the substitution of Na with Sr in bicine buffer for 1 step coating method resulted in a comparatively thinner pTAN coating.

An increased C 1s signal was detected on Ti-pTAN substrate. The observed carbon to oxygen ratio (C/O, 2.17) was slightly higher than theoretical C/O ratio of pTAN (1.65), which may be the result of carbon contamination. Nonetheless, for Ti-pTAN-1Stp (Figure 2C and Table 1) and Ti-pTAN-2Stp (Figure 2D and Table 1), the C/O ratios were 1.52 and 1.64 respectively. Moreover, distinct Sr 3d signals were observed on Ti-pTAN-1Stp and Ti-pTAN-2Stp, suggesting the successful immobilization of Sr by pTAN coating.

**Figure 3:**
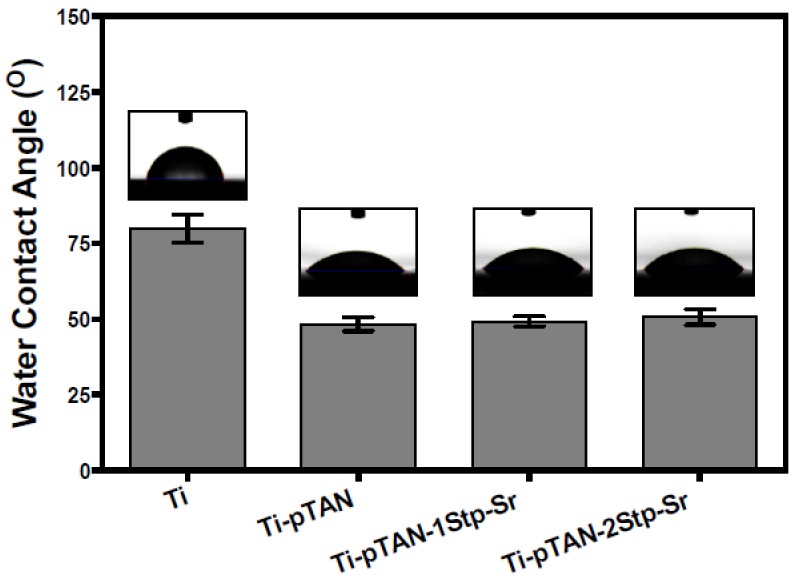
Contact angle analysis of Ti, Ti-pTAN, Ti-pTAN-1Stp-Sr and Ti-pTAN-2Stp-Sr.

The surface roughness of the coatings was comparable to Ti. The Ra values of Ti, Ti-pTAN, pTAN-1Stp-Sr and Ti-pTAN-2Stp-Sr were 0.44 ± 0.10 μm, 0.46 ± 0.05 μm, 0.46 ± 0.09 μm and 0.42 ± 0.06 μm, respectively. Water contact angle of the surface were examined. The contact angle of pristine Ti was 82° ± 0.9° (Figure 3). Hydrophilicity was conferred by pTAN coating, as contact was decreased to 48° ± 2.2° on Ti-pTAN. This is expected as multiple hydrophilic phenol moieties in pTAN increase wettability of the surface [28]. However, the water contact angles of Ti-pTAN-1Stp-Sr and Ti-pTAN-2Stp-Sr were 49° ± 1.7° and 51° ± 2.6° respectively, which were comparable to Ti-pTAN. Hence, Sr immobilization did not have any significant effect on contact angle of pTAN coating. Surface density of Sr on Ti-pTAN-1Stp-Sr and Ti-pTAN-2Stp-Sr determined ICP-MS were 0.32 ± 0.02 μg/cm^2^ and 5.8 ± 0.18 μg/cm^2^ respectively. 

### 3.2. Strontium Release

**Figure 4:**
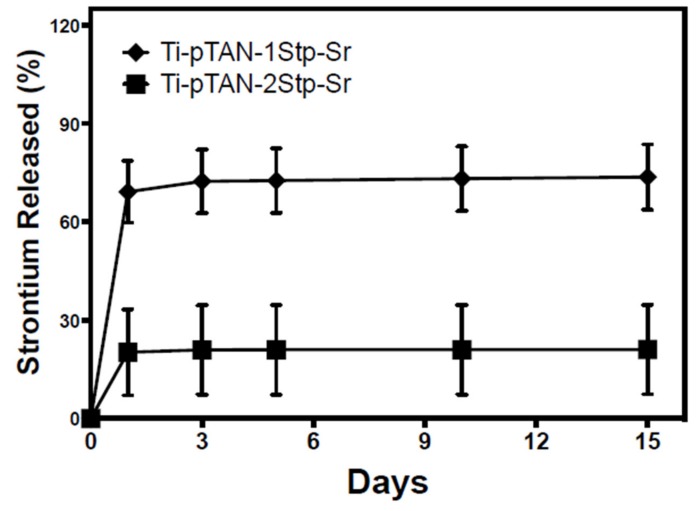
Sr release from Ti-pTAN-1Stp-Sr and Ti-pTAN-2Stp-Sr after incubation in PBS at 37 °C.

The release of Sr from Ti-pTAN-1Stp-Sr and Ti-pTAN-2Stp-Sr was estimated by ICP-MS. A burst release was observed after 24 h (Figure 4). However, subsequent time points revealed minimal release, with about 20% remaining on Ti-pTAN-1Stp-Sr and about 70% of the immobilized Sr remaining on Ti-pTAN-2Stp-Sr for 2 weeks. This shows that the binding of Sr^2+^ ions on Ti-pTAN-2Stp-Sr were stronger, compared to Ti-pTAN-1Stp-Sr. 

### 3.3. MC3T3-E1 Cell Viability

**Figure 5:**
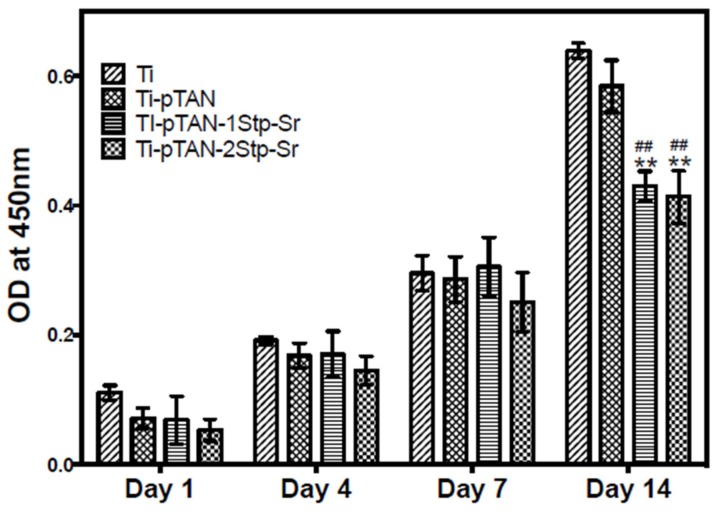
Proliferation study: CCK8 assay of MC3T3-E1 cells cultured on Ti, Ti-pTAN, Ti-pTAN-1Stp-Sr and Ti-pTAN-2Stp-Sr at various time points. (**) represents a statistically significant difference with *p* < 0.01 as compared to Ti and (**##**) represents the *p* < 0.01 as compared to Ti-pTAN for the same time point.

The cell viability of MC3T3-E1 cells on Ti, Ti-pTAN, Ti-pTAN-1Stp-Sr and Ti-pTAN-2Stp-Sr were monitored by CCK-8 assay kit. No difference in cell viability was observed for day 1, day 4 and day 7 of cell culture (Figure 5). However, a statistically significant decrease in cell metabolism was observed on day 14 for the MC3T3-E1 cells cultured on Ti-pTAN-1Stp-Sr and Ti-pTAN-2Stp-Sr as compared to Ti (*p* < 0.01) and Ti-pTAN (*p* < 0.01). 

### 3.4. Osteoblast Differentiation

**Figure 6:**
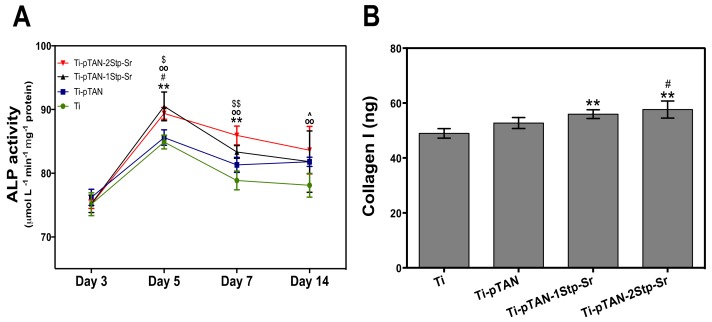
Early differentiation marker of MC3T3-E1: (**A**) ALP activity of MC3T3-E1 cells cultured on Ti, Ti-pTAN, Ti-pTAN-1Stp-Sr and Ti-pTAN-2Stp-Sr were studied on day 3, day 5, day 7 and day 14 of cell culture. Statistically significant differences are denoted as (**) *p* < 0.01 for Ti-pTAN-1Stp compared to Ti; (#) *p* < 0.05 for Ti-pTAN-1Stp compared to Ti-pTAN; (oo) for Ti-pTAN-2Stp compared to Ti; ($) *p* < 0.05 or ($$) *p* < 0.01 for Ti-pTAN-2Stp compared to Ti-pTAN and (^) *p* < 0.05 for Ti-pTAN compared to Ti. All the statistical comparisons were performed within the same time point; (**B**) Collagen 1 estimation was performed after 14 days of MC3T3-E1 culture on various substrates. Statistical significance is marked as (**) *p* < 0.01 as compared to Ti and (#) *p* < 0.05 as compare to Ti-pTAN.

The effects of the substrates on ALP activity of MC3T3-E1 cells were monitored at day 3, day 5, day 7 and day 14 of culture (Figure 6A). For day 3 cultures, ALP activity of osteoblasts on Ti was at a similar level as those on the other substrates. ALP values reached peak at day 5. Osteoblasts on Ti-pTAN-1Stp-Sr and Ti-pTAN-2Stp-Sr had significantly higher ALP activity as compared to Ti (*p* < 0.01) and pTAN (*p* < 0.05). From day 7 onwards, decrease in ALP activity was observed. Nevertheless, ALP activity was significantly higher on Ti-pTAN-1Stp-Sr as compared to Ti (*p* < 0.01), and for Ti-pTAN-2Stp-Sr, the activity was higher as compared to both Ti (*p* < 0.01) and Ti-pTAN (*p* < 0.01) at the same time point. ALP activity on day 14 was the lowest for all the substrates, but the activity was still higher on Ti-pTAN-2Stp-Sr (*p* < 0.01) and Ti-pTAN (*p* < 0.05) as compared to Ti. 

**Figure 7:**
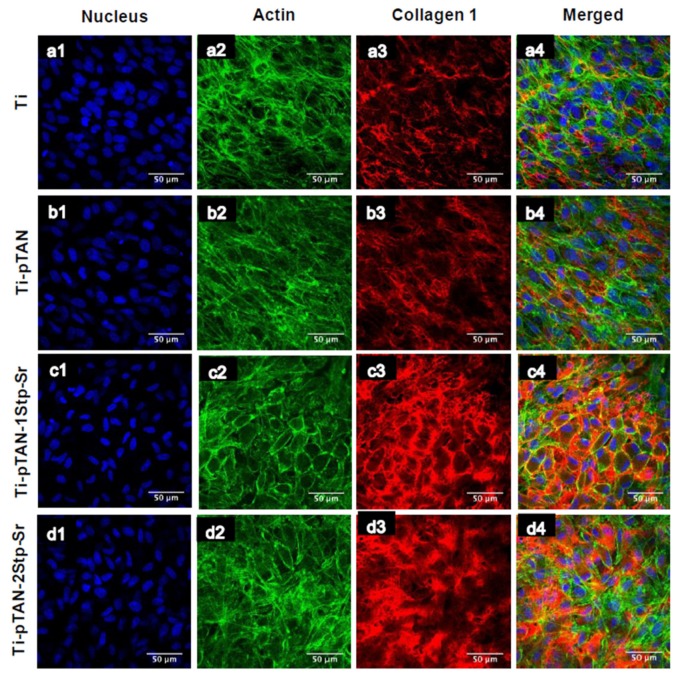
CSLM images of MC3T3-E1 cells after 21 days of culture on Ti (**a1**–**a4**); Ti-pTAN (**b1**–**b4**); Ti-pTAN-1Stp-Sr (**c1**–**c4**); Ti-pTAN-2Stp-Sr (**d1**–**d4**). MC3T3-E1 cells were stained for nucleus (**blue**, a1, b1, c1and d1); actin (**green**, a2, b2, c2and d2); collagen 1 (**red**, a3, b3, c3 and d3).

COLL 1 production of osteoblast cultured on Ti-pTAN-1Stp-Sr and Ti-pTAN-2Stp-Sr was statistically higher as compared to osteoblast cultured on Ti (*p* < 0.01, Figure 6B). Unlike the osteoblasts cultured on Ti-pTAN-1Stp-Sr, the osteoblasts cultured on Ti-pTAN-2Stp-Sr had higher COLL1 secretion as compared to Ti-pTAN (*p* < 0.05), suggesting superior osteogenic effects of Ti-pTAN-2Stp-Sr over Ti-pTAN-1Stp-Sr. The COLL 1 amounts were statistically similar for osteoblast cultured on Ti and Ti-pTAN. Immunofluorescence labelling of cells further confirmed these results for COLL 1. After 21 days of culture, MC3T3-E1 cells were stained for COLL1 using anti-COLL 1 antibody (Figure 7). MC3T3-E1 cells cultured on Ti-pTAN-1Stp-Sr (Figure 7c3) and Ti-pTAN-2Stp-Sr (Figure 6d3) displayed higher COLL 1 production as compared to cells cultured on Ti (Figure 7a3) and Ti-pTAN (Figure 7b3). Actin staining was also performed for the cells (Figure 7a2,b2,c2,d2) to monitor cell morphology and density. The cells cultured on all the substrates were confluent with uniform cell density. Cuboidal morphology was observed for the cells cultured on Ti-pTAN-1Stp-Sr and Ti-pTAN-2Stp-Sr. But the cells on Ti and Ti-pTAN had fusiform morphologies. 

**Figure 8:**
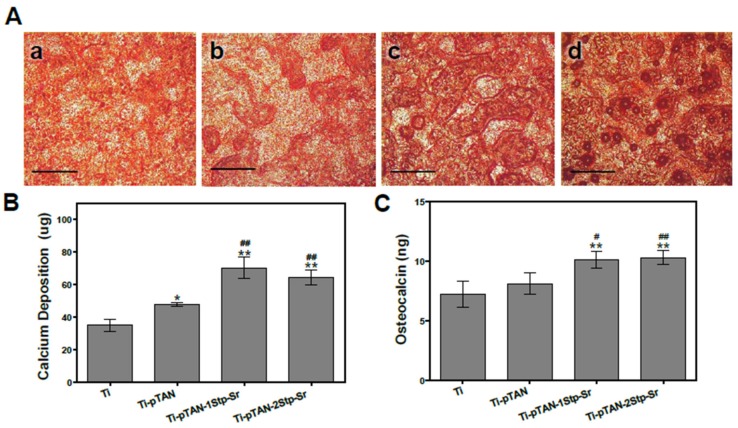
Calcium deposition and osteocalcin production of MC3T3-E1 cells after 14 days of culture on various substrates. (**A**) Alizarin S stain of calcium deposited by MC3T3-E1 cell on Ti (**a**); Ti-pTAN (**b**); Ti-pTAN-1Stp-Sr (**c**) and Ti-pTAN-2Stp-Sr (**d**); Scale bar 200 μm. (**B**) Estimation of calcium deposition by MC3T3-E1 cells; (**C**) Osteocalcin production by MC3T3-E1 cells cultured on the substrates. Statistical significance is represented as (*) *p* < 0.05 or (**) *p* < 0.01 as compared to Ti and (#) *p* < 0.05 or (##) *p* < 0.01 as compared to Ti-pTAN.

The effect of immobilized Sr on late differentiation markers such as osteocalcin [29] and matrix mineralization were investigated after 14 days of culture. Alizarin S stain and calcium estimation were performed to monitor the effects of Sr immobilized substrates on osteoblast mineralization. Stronger alizarin stain was observed on Ti-pTAN-1Stp-Sr (Figure 8Ac) and Ti-pTAN-2Stp-Sr (Figure 8Ad) as compared to Ti (Figure 7Aa) and Ti-pTAN (Figure 7Ab). Similarly, calcium estimated on Ti-pTAN-1Stp-Sr and Ti-pTAN-2Stp-Sr was higher than on Ti (*p* < 0.01) and Ti-pTAN (*p* < 0.01) (Figure 8B). Moreover, calcium amounts on Ti-pTAN were elevated as compared to Ti (*p* < 0.05), suggesting that pTAN coatings have a positive influence on osteoblast mineralization. Osteocalcin production was also higher on Ti-pTAN-1Stp-Sr and Ti-pTAN-2Stp-Sr as compared to Ti (*p* < 0.01) and Ti-pTAN (*p* < 0.05 and *p* < 0.01, respectively). 

### 3.5. Osteoclast Differentiation

**Figure 9:**
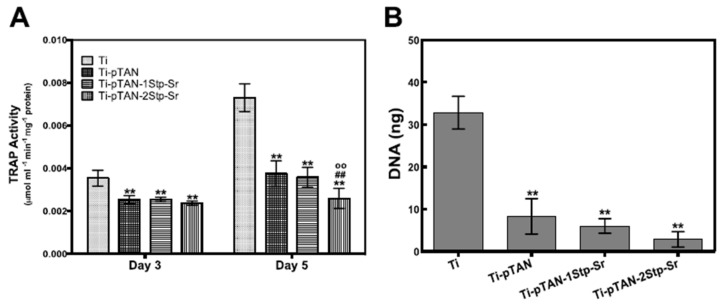
Osteoclast TRAP activity (**A**) and total DNA; (**B**) after 5 days of culture on Ti, Ti-pTAN, Ti-pTAN-1Stp-Sr, Ti-pTAN-2Stp-Sr.

TRAP activity was estimated after day 3 and day 5 of culture in cell culture supernatant. For day 3 cultures, TRAP activity was reduced on Ti-pTAN, Ti-pTAN-1Stp-Sr and Ti-pTAN-2Stp-Sr as compared to Ti (*p* < 0.01) (Figure 9A). This suggested that the decrease of TRAP on all the substrates was due to pTAN coating. On day 5, cells on Ti-pTAN, Ti-pTAN-1Stp-Sr and Ti-pTAN-2Stp-Sr substrates also showed a reduction in TRAP as compared to Ti (*p* < 0.01). TRAP activity on Ti-pTAN-2Stp-Sr was even lower than cells on Ti-pTAN (*p* < 0.01), Ti-pTAN-1Stp-Sr (*p* < 0.01). This suggested that Sr immobilized on Ti-pTAN-2Stp-Sr with higher surface density has superior inhibitory effects towards osteoclast differentiation as compared to Ti-pTAN-1Stp-Sr. Total cell DNA estimation was used to study cell proliferation as per previous reports [30,31]. Cell proliferation after 5 days of RANKL stimulation of RAW 264.7 was also monitored. Total DNA was significantly reduced on Ti-pTAN, Ti-pTAN-1Stp-Sr and Ti-pTAN-2Stp-Sr as compared to Ti (*p* < 0.01) (Figure 9B).

**Figure 10:**
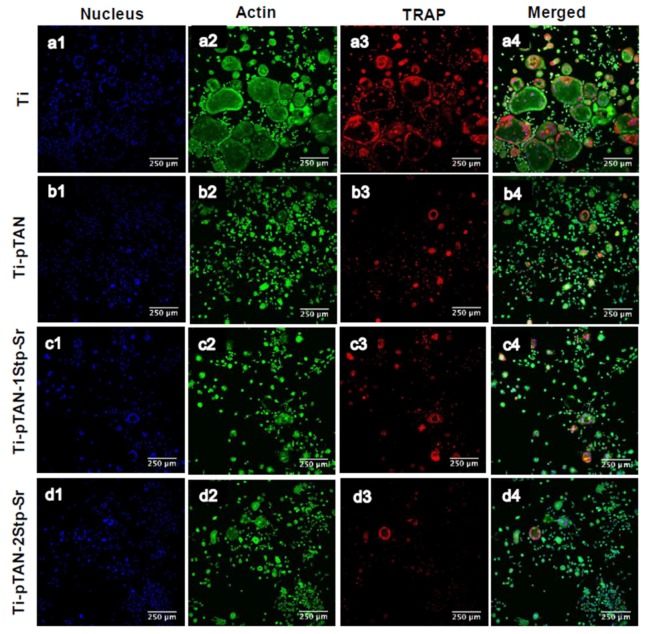
CSLM images of RAW 264.7 cells after 5 days of culture in presence of RANKL. Cells were cultured on Ti (**a1**–**a4**), Ti-pTAN (**b1**–**b4**), Ti-pTAN-1Stp-Sr (**c1**–**c4**), Ti-pTAN-2Stp-Sr (**d1**–**d4**) and were stained for nucleus (**blue**, a1, b1, c1and d1), actin (**green**, a2, b2, c2and d2), TRAP (**red**, a3, b3, c3 and d3).

Cells after 5 days of culture on Ti, Ti-pTAN, Ti-pTAN-1Stp-Sr and Ti-pTAN-2Stp-Sr were stained for actin and TRAP (Figure 10). TRAP positive multi-nucleated cells with more than 3 nuclei were considered as osteoclasts. Unlike Ti (Figure 10a1–4), which displayed large multinucleated TRAP positive osteoclasts, smaller and reduced number of multinucleated TRAP positive cells were observed on Ti-pTAN (Figure 9b1–4), Ti-pTAN-1Stp-Sr (Figure 10c1–4) and Ti-pTAN-2Stp-Sr (Figure 10d1–4). Similar to the TRAP activity estimation data of cell culture supernatant, intracellular TRAP production was decreased for cells on Ti-pTAN (Figure 10b3), Ti-pTAN-1Stp-Sr (Figure 9c3) and Ti-pTAN-2Stp-Sr (Figure 9d3) as compared to Ti (Figure 10a3). Cells especially on Ti-pTAN-2Stp-Sr depicted the lowest TRAP production of all the substrates. This suggested that the osteoclast inhibitory effects of Ti-pTAN-2Stp-Sr were superior to both Ti-pTAN-1Stp-Sr and Ti-pTAN.

## 4. Discussion

Since the imbalance in osteoclast and osteoblast activity in patients with bone metabolic disease can be an obstacle to bone implant success, coatings that actively aim at correcting this imbalance are required to recover bone turnover and improve the osseointegration of bone implants. In this study, a pTAN coating was used to immobilize Sr on a Ti surface. Two separate schemes were employed (Figure 1); the first scheme was a one-step process, wherein Ti-pTAN-1Stp-Sr substrates were prepared by replacing sodium with Sr in tannic acid solution in bicine buffer. In the second scheme, Ti-pTAN-2Stp-Sr substrates were prepared by a two-step method, wherein a pTAN coating was carried out in a bicine buffer containing sodium, before coating the substrates with Sr. The Sr metal ions were immobilized on pTAN-coated Ti (Figure 2 and Table 1). The surface immobilization efficiency was higher for the two-step coating method. Hence, unlike the one-step coating method, the two-step coating method not only displayed higher surface density of immobilized Sr, but also showed stronger binding, as 70% of the Sr remained bound and was not released for two weeks (Figure 4). The thicker tannic acid coating conferred by the two-step method could contribute to more phenol groups increasing their interactions with Sr^2+^ ions to increase immobilization efficiency. pTAN coating kinetics and mechanisms have been described previously [32]. It is proposed that the first layer of pTAN on the Ti surface is though the formation of TiO(OH)pTAN hydroxo complexes [25,32]. Subsequent layers are built up by the heterogeneous polymers formed by the autooxidation of pTAN [25]. pTAN is also known to form polymers through the formation of multivalent coordination bonding with metal ions [24,26]. Hence, it is assumed that the pTAN-Sr coatings on Ti-pTAN-1Stp-Sr and Ti-pTAN-2Stp-Sr are formed by auto-oxidation of pTAN and multivalent coordination bonding with Sr.

A decrease in osteoblast proliferation was observed for cells cultured on Ti-pTAN-1Stp-Sr and Ti-pTAN-2Stp-Sr (Figure 5). Joseph Caverzasio observed that the addition of Sr to a cell culture medium enhanced osteoblast proliferation [3]. Almeida et al. reported that a Sr salt supplemented in media did not affect MC3T3-E1 proliferation at lower concentrations (0.05 and 1 mM), but at higher concentrations such as 0.5 mM, a decrease in proliferation was observed as compared to that of untreated controls [33,34]. Furthermore, Ti functionalized by Sr via magnetron co-sputtering technology also decreased proliferation of human dental pulp stem cells. [19] Yet, in both studies, osteoblast differentiation was augmented due to Sr introduction, which was consistent with our results, as described below. It is known that a decrease in osteoblast proliferation and growth arrest are associated with the elevated production of differentiation markers such as ALP, COLL 1 etc. [35,36] As such, the effects of Ti-pTAN-1Stp-Sr and Ti-pTAN-2Stp-Sr on osteoblast differentiation markers were investigated. 

ALP and COLL 1 are early differentiation markers for osteoblasts, whereas osteocalcin is a late differentiation marker [29]. By secreting ALP and collagen 1 in the early phases of differentiation and mineralizing matrix in the later stages, MC3T3-E1 exhibits successive differentiation sequence which is comparable to osteoblast in bone [35]. Hence, this cell line is widely used as in vitro model for osteoblasts research. Generally, the ALP expression of differentiating osteoblasts augments in the early phases of differentiation, leading to matrix maturation, while the level drops before matrix mineralization [37]. As presented, ALP production was enhanced on day 5 for all the substrates with a decline in the activity on day 7 and day 14, suggesting the progression of the cells to the mineralization phase (Figure 6A). Yet, compared to Ti, Sr-immobilized substrates such as Ti-pTAN-1Stp-Sr and Ti-pTAN-2Stp-Sr boosted ALP activity on day 5 and day 7. COLL 1 secretion, which is also an early differentiation marker of osteoblasts, was monitored after 14 days of culture (Figure 6B and Figure 7) [29]. Sr surface coated substrates displayed higher COLL 1 production. The majority of MC3T3-E1 cells on Sr immobilized substrates, in particular the cells on Ti-pTAN-1Stp-Sr, exhibited cuboidal morphology (Figure 6), which is a characteristic feature of differentiating cells [38]. On the other hand, the majority of cells cultured on Ti and Ti-pTAN displayed a fusiform appearance (Figure 6), which is a predominant feature of proliferating cells [38]. This further corroborates the osteogenic potential of Sr immobilized substrates. Furthermore, the Sr-based surface coatings also augmented in the osteocalcin and matrix mineralization by the osteoblasts (Figure 8). 

Studies had shown that strontium supplementation in media inhibited osteoblast mineralization in vitro [39,40]. Contrastingly, another report established that Sr enhanced ALP, osteocalcin secretion and the mineralization of mouse bone marrow stromal cells [41], and human primary osteoblasts [42]. Sr supplemented in cell culture media as a SrCl_2_ triggered osteogenic differentiation of human mesenchymal stem cells (MSCs) and stimulated in vivo bone deposition when delivered locally as Sr-HA-collagen scaffold to treat rat calvarial defect via Wnt signal transduction [43]. In this study, Sr-immobilized substrates such as Ti-pTAN-1Stp-Sr and Ti-pTAN-2Stp-Sr augmented ALP activity, COLL 1 production, osteocalcin secretion and calcium deposition of MC3T3-E1 cells. However, Ti-pTAN-2Stp-Sr showed a higher surface density of Sr as compared to Ti-pTAN-1Stp-Sr, although the potential to augment osteogenesis was similar for both the substrates. This suggests that even if the two-step method of coating resulted in a higher efficiency of Sr binding properties, a low Sr density on the surface, such as 0.32 ± 0.02 μg/cm^2^, is sufficient in promoting osteoblast differentiation.

Osteoclasts are important cells for bone remodeling [44]; hence, the regulation of their activity on implant surfaces is crucial for osseointegration. The effects of osteoclasts on Sr-modified surfaces were also investigated. As a RANKL-sensitive pre-osteoclast population, RAW 264.7 can be easily procured and is easy to handle as compared to primary cultures, which makes is an attractive in vitro model for osteoclast research [45]. Osteoclastic differentiation of RAW 264.7 cells on Ti, Ti-pTAN, Ti-pTAN-1Stp-Sr and Ti-pTAN-2Stp-Sr were studied by estimating TRAP activity. TRAP activity was used as a marker for osteoclast differentiation and resorption [46,47,48]. pTAN coated substrates such as Ti-pTAN, Ti-pTAN-1Stp-Sr and Ti-pTAN-2Stp-Sr decreased TRAP activity and total DNA (Figure 9). However, on day 5, the decrease in TRAP production was greatest in Ti-pTAN-2Stp-Sr. Likewise, immunofluorescence labelling of intracellular TRAP production was also lowest on the Ti-pTAN-2Stp-Sr substrate (Figure 10d3). Strontium ranelate was shown to inhibit RANKL mediated osteoclastogenesis of RAW 264.7 and human peripheral blood monocyte cells [49]. As shown by Chung et al., Ti modified with Sr substituted HA by micro-arc treatment reduced osteoclast differentiation of RAW 264.7 cells [50]. Equally, this study demonstrated that Ti-pTAN-2Stp-Sr reduced osteoclast activity as compared to Ti and Ti-pTAN. Florencio-Silva et al. reported that osteoclast precursors fuse, polarize, and rearrange actin cytoskeleton to form actin rings and a sealing zone [51]. The formation of multinucleated cells was also reduced on all the pTAN coated substrates such as Ti-pTAN, Ti-pTAN-1Stp-Sr and Ti-pTAN-2Stp-Sr (Figure 10). However, the inhibitory effects of Ti-pTAN-2Stp-Sr were superior to those of Ti, Ti-pTAN and Ti-pTAN-1Stp-Sr substrates.

## 5. Conclusions

In this study, a pTAN coating was used to immobilize Sr on a Ti surface. Although Ti-pTAN-2Stp-Sr showed a higher surface density of Sr as compared to Ti-pTAN-1Stp-Sr, the ability to augment osteogenesis was similar for both the substrates. Osteoclast differentiation studies revealed that the Ti-pTAN-2Stp-Sr substrates decreased the TRAP activity of osteoclast significantly as compared to Ti, Ti-pTAN and Ti-pTAN-1Stp-Sr. The pTAN coating also had an influence on the negative regulation of osteoclast differentiation, as reported [52]. Henceforth, this economical and simple coating of pTAN with inherent anti-osteoclastogenic properties could be exploited to immobilize osteogenic metal ions such as Sr^2+^ on surfaces. The two-step method was more efficient to immobilize Sr, as well as to modulate osteoblast and osteoclast cells. 

It is hoped that these findings will be beneficial to deliver Sr in order to tailor cellular responses at bone-implant interfaces, especially for osteoporotic bone. The imbalance in osteoclasts and osteoblasts activity in patients with bone metabolic disease such as osteoporosis can be an obstacle to bone implant success [53,54,55]. Coatings that actively aim at correcting this imbalance are required to recover bone turnover and improve the osseointegration of bone implants [56,57]. We believe that these surface coatings will be beneficial to regulate osteoclast and osteoblast development at implant surfaces. Nonetheless, we acknowledge that the in vitro results presented here are conducted in controlled laboratory settings and could not be extrapolated to in vivo or clinical conditions. The coupling of bone formation and bone resorption in a basic multicellular unit of a healthy bone is a multifaceted process which involves the interplay of various regulatory molecules and bone cells [58,59]. Further studies are needed to understand the viability of coatings and the potential to regulate the molecular and cellular interplay in in vivo settings.

## Figures and Tables

**Table 1:** Elemental composition as percentages ^a^ assessed by XPS.

Substrates	C%	O%	Ti%	Sr%
Ti	38.11	50.75	11.14	0
Ti-pTAN	66.03	30.43	3.54	0
Ti-pTAN-1Stp	55.49	36.38	7.68	0.45
Ti-pTAN-2Stp	58.84	35.8	4.47	0.89

^a^ Percentage estimation of elements were based on C, O, Ti and Sr only.

## References

[1] Marcus R. (2002). Post-menopausal osteoporosis. Best Pract. Res. Clin. Obstet. Gynaecol..

[2] Sessa G., Evola F.R., Costarella L. (2011). Osteosynthesis systems in fragility fracture. Aging Clin. Exp. Res..

[3] Junker R., Dimakis A., Thoneick M., Jansen J.A. (2009). Effects of implant surface coatings and composition on bone integration: A systematic review. Clin. Oral Implant. Res..

[4] Palmquist A., Omar O.M., Esposito M., Lausmaa J., Thomsen P. (2010). Titanium oral implants: Surface characteristics, interface biology and clinical outcome. J. R. Soc. Interface.

[5] O’Brien E.M., Risser G.E., Spiller K.L. (2019). Sequential drug delivery to modulate macrophage behavior and enhance implant integration. Adv. Drug Deliv. Rev..

[6] Moroni A., Faldini C., Marchetti S., Manca M., Consoli V., Giannini S. (2001). Improvement of the bone-pin interface strength in osteoporotic bone with use of hydroxyapatite-coated tapered external-fixation pins. J. Bone J. Surg Am..

[7] Marchisio M., Carmine M.D., Pagone R., Piattelli A., Miscia S. (2005). Implant surface roughness influences osteoclast proliferation and differentiation. J. Biomed. Mater. Res. B Appl. Biomater..

[8] Schwartz Z., Lohmann C., Oefinger J., Bonewald L., Dean D., Boyan B. (1999). Implant surface characteristics modulate differentiation behavior of cells in the osteoblastic lineage. Adv. Dent. Res..

[9] Klymov A., Prodanov L., Lamers E., Jansen J.A., Walboomers X.F. (2013). Understanding the role of nano-topography on the surface of a bone-implant. Biomater. Sci..

[10] Poh C.K., Shi Z., Lim T.Y., Neoh K.G., Wang W. (2010). The effect of VEGF functionalization of titanium on endothelial cells in vitro. Biomaterials.

[11] Tan H.C., Poh C.K., Cai Y., Soe M.T., Wang W. (2013). Covalently grafted BMP-7 peptide to reduce macrophage/monocyte activity: An in vitro study on cobalt chromium alloy. Biotechnol. Bioeng..

[12] Sachse A., Wagner A., Keller M., Wagner O., Wetzel W.D., Layher F., Venbrocks R.A., Hortschansky P., Pietraszczyk M., Wiederanders B. (2005). Osteointegration of hydroxyapatite-titanium implants coated with nonglycosylated recombinant human bone morphogenetic protein-2 (BMP-2) in aged sheep. Bone.

[13] Gao Y., Zou S., Liu X., Bao C., Hu J. (2009). The effect of surface immobilized bisphosphonates on the fixation of hydroxyapatite-coated titanium implants in ovariectomized rats. Biomaterials.

[14] Lenart B.A., Lorich D.G., Lane J.M. (2008). Atypical Fractures of the Femoral Diaphysis in Postmenopausal Women Taking Alendronate. N. Engl. J. Med..

[15] Reginster J.-Y., Kaufman J.-M., Goemaere S., Devogelaer J.P., Benhamou C.L., Felsenberg D., Diaz-Curiel M., Brandi M.-L., Badurski J., Wark J. (2012). Maintenance of antifracture efficacy over 10 years with strontium ranelate in postmenopausal osteoporosis. Osteoporos. Int..

[16] Cianferotti L., D’Asta F., Brandi M.L. (2013). A review on strontium ranelate long-term antifracture efficacy in the treatment of postmenopausal osteoporosis. Ther. Adv. Musculoskelet. Dis..

[17] Bain S.D., Jerome C., Shen V., Dupin-Roger I., Ammann P. (2009). Strontium ranelate improves bone strength in ovariectomized rat by positively influencing bone resistance determinants. Osteoporos. Int..

[18] Bonnelye E., Chabadel A., Saltel F., Jurdic P. (2008). Dual effect of strontium ranelate: Stimulation of osteoblast differentiation and inhibition of osteoclast formation and resorption in vitro. Bone.

[19] Andersen O.Z., Offermanns V., Sillassen M., Almtoft K.P., Andersen I.H., Sørensen S., Jeppesen C.S., Kraft D.C.E., Bøttiger J., Rasse M. (2013). Accelerated bone ingrowth by local delivery of strontium from surface functionalized titanium implants. Biomaterials.

[20] Tao Z.S., Bai B.L., He X.W., Liu W., Li H., Zhou Q., Sun T., Huang Z.L., Tu K.K., Lv Y.X. (2016). A comparative study of strontium-substituted hydroxyapatite coating on implant’s osseointegration for osteopenic rats. Med. Biol. Eng. Comput..

[21] Dang Y., Zhang L., Song W., Chang B., Han T., Zhang Y., Zhao L. (2016). In vivo osseointegration of Ti implants with a strontium-containing nanotubular coating. Int. J. Nanomed..

[22] Tao Z.S., Zhou W.S., He X.W., Liu W., Bai B.L., Zhou Q., Huang Z.L., Tu K.K., Li H., Sun T. (2016). A comparative study of zinc, magnesium, strontium-incorporated hydroxyapatite-coated titanium implants for osseointegration of osteopenic rats. Mater. Sci. Eng. C Mater. Biol. Appl..

[23] Sileika T.S., Barrett D.G., Zhang R., Lau K.H., Messersmith P.B. (2013). Colorless multifunctional coatings inspired by polyphenols found in tea, chocolate, and wine. Angew. Chem..

[24] Kim S., Pasc A. (2017). Advances in Multifunctional Surface Coating Using Metal-Phenolic Networks. Bull. Korean Chem. Soc..

[25] Geissler S., Barrantes A., Tengvall P., Messersmith P.B., Tiainen H. (2016). Deposition Kinetics of Bioinspired Phenolic Coatings on Titanium Surfaces. Langmuir.

[26] Ejima H., Richardson J.J., Liang K., Best J.P., van Koeverden M.P., Such G.K., Cui J., Caruso F. (2013). One-step assembly of coordination complexes for versatile film and particle engineering. Science.

[27] Robinson K.S., Sherwood P. (1984). X-ray photoelectron spectroscopic studies of the surface of sputter ion plated films. Surf. Interface Anal..

[28] Pan L., Wang H., Wu C., Liao C., Li L. (2015). Tannic-Acid-Coated Polypropylene Membrane as a Separator for Lithium-Ion Batteries. ACS Appl. Mater. Interfaces.

[29] Huang W., Yang S., Shao J., Li Y.-P. (2007). Signaling and transcriptional regulation in osteoblast commitment and differentiation. Front. Biosci..

[30] Jones G.L., Motta A., Marshall M.J., El Haj A.J., Cartmell S.H. (2009). Osteoblast: Osteoclast co-cultures on silk fibroin, chitosan and PLLA films. Biomaterials.

[31] Rose F.R., Cyster L.A., Grant D.M., Scotchford C.A., Howdle S.M., Shakesheff K.M. (2004). In vitro assessment of cell penetration into porous hydroxyapatite scaffolds with a central aligned channel. Biomaterials.

[32] Surleva A., Atanasova P., Kolusheva T., Costadinnova L. (2014). Study of the complex equilibrium between titanium (IV) and tannic acid. J. Chem. Technol. Metall..

[33] Caverzasio J. (2008). Strontium ranelate promotes osteoblastic cell replication through at least two different mechanisms. Bone.

[34] Almeida M.M., Nani E.P., Teixeira L.N., Peruzzo D.C., Joly J.C., Napimoga M.H., Martinez E.F. (2016). Strontium ranelate increases osteoblast activity. Tissue Cell.

[35] Quarles L.D., Yohay D.A., Lever L.W., Caton R., Wenstrup R.J. (1992). Distinct proliferative and differentiated stages of murine MC3T3-E1 cells in culture: An in vitro model of osteoblast development. J. Bone Miner. Res. Off. J. Am. Soc. Bone Mineral. Res..

[36] Gu Y.X., Du J., Si M.S., Mo J.J., Qiao S.C., Lai H.C. (2013). The roles of PI3K/Akt signaling pathway in regulating MC3T3-E1 preosteoblast proliferation and differentiation on SLA and SLActive titanium surfaces. J. Biomed. Mater. Res. Part A.

[37] Lian J.B., Stein G.S. (1992). Concepts of osteoblast growth and differentiation: Basis for modulation of bone cell development and tissue formation. Crit. Rev. Oral Biol. Med..

[38] Hong D., Chen H.-X., Yu H.-Q., Liang Y., Wang C., Lian Q.-Q., Deng H.-T., Ge R.-S. (2010). Morphological and proteomic analysis of early stage of osteoblast differentiation in osteoblastic progenitor cells. Exp. Cell Res..

[39] Verberckmoes S.C., De Broe M.E., D’Haese P.C. (2003). Dose-dependent effects of strontium on osteoblast function and mineralization. Kidney Int..

[40] Wornham D.P., Hajjawi M.O., Orriss I.R., Arnett T.R. (2014). Strontium potently inhibits mineralisation in bone-forming primary rat osteoblast cultures and reduces numbers of osteoclasts in mouse marrow cultures. Osteoporos. Int..

[41] Choudhary S., Halbout P., Alander C., Raisz L., Pilbeam C. (2007). Strontium ranelate promotes osteoblastic differentiation and mineralization of murine bone marrow stromal cells: Involvement of prostaglandins. J. Bone Mineral. Res..

[42] Atkins G., Welldon K., Halbout P., Findlay D. (2009). Strontium ranelate treatment of human primary osteoblasts promotes an osteocyte-like phenotype while eliciting an osteoprotegerin response. Osteoporos. Int..

[43] Yang F., Yang D., Tu J., Zheng Q., Cai L., Wang L. (2011). Strontium enhances osteogenic differentiation of mesenchymal stem cells and in vivo bone formation by activating Wnt/catenin signaling. Stem Cells.

[44] Sims N.A., Gooi J.H. (2008). Bone remodeling: Multiple cellular interactions required for coupling of bone formation and resorption. Semin. Cell Dev. Biol..

[45] Collin-Osdoby P., Osdoby P. (2012). RANKL-mediated osteoclast formation from murine RAW 264.7 cells. Methods Mol. Biol..

[46] Kirstein B., Chambers T.J., Fuller K. (2006). Secretion of tartrate-resistant acid phosphatase by osteoclasts correlates with resorptive behavior. J. Cell. Biochem..

[47] Halleen J.M., Alatalo S.L., Suominen H., Cheng S., Janckila A.J., Väänänen H.K. (2000). Tartrate-resistant acid phosphatase 5b: A novel serum marker of bone resorption. J. Bone Mineral. Res..

[48] Lv Y., Wang G., Xu W., Tao P., Lv X., Wang Y. (2015). Tartrate-resistant acid phosphatase 5b is a marker of osteoclast number and volume in RAW 264.7 cells treated with receptor-activated nuclear κB ligand. Exp. Ther. Med..

[49] Caudrillier A., Hurtel-Lemaire A.-S., Wattel A., Cournarie F., Godin C., Petit L., Petit J.-P., Terwilliger E., Kamel S., Brown E.M. (2010). Strontium ranelate decreases receptor activator of nuclear factor-κB ligand-induced osteoclastic differentiation in vitro: Involvement of the calcium-sensing receptor. Mol. Pharmacol..

[50] Chung C.-J., Long H.-Y. (2011). Systematic strontium substitution in hydroxyapatite coatings on titanium via micro-arc treatment and their osteoblast/osteoclast responses. Acta Biomater..

[51] Florencio-Silva R., Sasso G.R., Sasso-Cerri E., Simoes M.J., Cerri P.S. (2015). Biology of Bone Tissue: Structure, Function, and Factors That Influence Bone Cells. BioMed Res. Int..

[52] Steffi C., Shi Z., Kong Chee H., Wang W. (2019). Bioinspired polydopamine and polyphenol tannic acid functionalized titanium suppress osteoclast differentiation: A facile and efficient strategy to regulate osteoclast activity at bone–implant interface. J. R. Soc. Interface.

[53] Schulze M., Gehweiler D., Riesenbeck O., Wähnert D., Raschke M.J., Hartensuer R., Vordemvenne T. (2017). Biomechanical characteristics of pedicle screws in osteoporotic vertebrae—Comparing a new cadaver corpectomy model and pure pull-out testing. J. Orthop. Res..

[54] Apostu D., Lucaciu O., Berce C., Lucaciu D., Cosma D. (2017). Current methods of preventing aseptic loosening and improving osseointegration of titanium implants in cementless total hip arthroplasty: A review. J. Int. Med. Res..

[55] Weiser L., Huber G., Sellenschloh K., Viezens L., Püschel K., Morlock M.M., Lehmann W. (2017). Insufficient stability of pedicle screws in osteoporotic vertebrae: Biomechanical correlation of bone mineral density and pedicle screw fixation strength. Eur. Spine J..

[56] Civantos A., Martínez-Campos E., Ramos V., Elvira C., Gallardo A., Abarrategi A. (2017). Titanium Coatings and Surface Modifications: Toward Clinically Useful Bioactive Implants. ACS Biomater. Sci. Eng..

[57] Zhao H., Huang Y., Zhang W., Guo Q., Cui W., Sun Z., Eglin D., Liu L., Pan G., Shi Q. (2018). Mussel-Inspired Peptide Coatings on Titanium Implant to Improve Osseointegration in Osteoporotic Condition. ACS Biomater. Sci. Eng..

[58] Sims N.A., Martin T.J. (2014). Coupling the activities of bone formation and resorption: A multitude of signals within the basic multicellular unit. Bonekey Rep..

[59] Kohli N., Ho S., Brown S.J., Sawadkar P., Sharma V., Snow M., García-Gareta E. (2018). Bone remodelling in vitro: Where are we headed? A review on the current understanding of physiological bone remodelling and inflammation and the strategies for testing biomaterials in vitro. Bone.

